# Segmentation of Oil Spills on Side-Looking Airborne Radar Imagery with Autoencoders

**DOI:** 10.3390/s18030797

**Published:** 2018-03-06

**Authors:** Antonio-Javier Gallego, Pablo Gil, Antonio Pertusa, Robert B. Fisher

**Affiliations:** 1 Pattern Recognition and Artificial Intelligence Group, Department of Software and Computing Systems, University of Alicante, E-03690 Alicante, Spain; pertusa@dlsi.ua.es; 2 Automation, Robotics and Computer Vision Group, Department of Physics, Systems Engineering and Signal Theory, University of Alicante, E-03690 Alicante, Spain; pablo.gil@ua.es; 3 Computer Science Research Institute, University of Alicante, E-03690 Alicante, Spain; 4 School of Informatics, University of Edinburgh, EH1 2QL Edinburgh, UK; rbf@inf.ed.ac.uk

**Keywords:** oil spill detection, side-looking airborne radar, neural networks, supervised learning, radar detection

## Abstract

In this work, we use deep neural autoencoders to segment oil spills from Side-Looking Airborne Radar (SLAR) imagery. Synthetic Aperture Radar (SAR) has been much exploited for ocean surface monitoring, especially for oil pollution detection, but few approaches in the literature use SLAR. Our sensor consists of two SAR antennas mounted on an aircraft, enabling a quicker response than satellite sensors for emergency services when an oil spill occurs. Experiments on TERMA radar were carried out to detect oil spills on Spanish coasts using deep selectional autoencoders and RED-nets (very deep Residual Encoder-Decoder Networks). Different configurations of these networks were evaluated and the best topology significantly outperformed previous approaches, correctly detecting 100% of the spills and obtaining an $F_{1}$ score of 93.01% at the pixel level. The proposed autoencoders perform accurately in SLAR imagery that has artifacts and noise caused by the aircraft maneuvers, in different weather conditions and with the presence of look-alikes due to natural phenomena such as shoals of fish and seaweed.

## 1. Introduction

A quick response from governments is required in situations of marine pollution due to oil spills [1]. When an oil slick is detected, the authorities activate the emergency protocols in order to control the environmental impact and the ecological damage in the sea. The most relevant technologies and spaceborne sensors for oil-spill sensing are described in [2,3,4]. CleanSeaNet is an example of a monitoring service of oil spills and vessels provided by the European Maritime Safety Agency (EMSA). Governments use mainly two kinds of sensors to carry out the monitoring of the sea surface: Synthetic Aperture Radar (SAR) installed on satellites (ERS-1/2, JERS-1, Envisat ASAR, RADARSAT-1, RADARSAT-2, COSMO-SkyMed, Sentinel-1, Sentinel-2, ALOS-2, TerraSAR-X among others) as in CleanSeaNet, and Side-Looking Airborne Radar (SLAR) or another airborne miniaturized radar as in [5]. Both sensors can be used for oil slick detection.

The SLAR used in this work is a SAR mounted on an aircraft instead of a satellite and it has two radar antennas. SLAR and SAR sensors have some differences as mentioned in [6]. On the one hand, SLAR has a range and resolution smaller than SAR and, consequently, the complexity in the detection is higher due to the lower details in the acquired image. However, SLAR does not depend on the orbit because it is mounted on an aircraft, and therefore it has a better response time than SAR. As aircraft can modify their altitude and flight path during signal acquisition, SLAR images have different perspectives and scale. In addition, these images have artifacts and noisy areas caused by the aircraft motion (turns, slips, etc.) and by the location of the two SLAR antennas under the aircraft wings. These artifacts and types of noise are not present in SAR images in which speckle (with granular appearance) is the most common noise.

The oil-spill detection strategies using SAR can be categorized into two groups. The first contains all the approaches that use the raw signals of the radar as well as polarimetric (PolSAR) or interferometric features (InSAR), and so forth to discriminate the oil slicks [7,8]. The second includes the methods that use intensity images as a representation of the backscattering coefficient of the signal [9,10]. In addition, some works such as [11] combine image and polarimetric features extracted from oil spills and look-alikes in order to discriminate between both targets.

In the state of the art, there are many works which address oil spill detection using machine learning techniques. These methods include Tree Forests [12], Support Vector Machines (SVM) [13,14], Generalized Linear Models (GLM) and Boosting trees, among others, as in [15,16], where both a Bayesian classifier and several evolutionary algorithms were used to select image features for classifying oil spills and look-alikes. Neural networks have also been used for this task, using as input different features from radar images characterizing an candidate oil slick [17,18]. The choice of the classifier architecture is dependent on the problem and when the features are properly selected there are no significant differences in the results, as shown in [19] with PolSAR data.

In some previous works, image processing and computer vision algorithms were used to automatically extract features and segment regions from radar images. These data can be fed to a network such as in [20], in which two neural networks were proposed, one to detect dark formations and another to classify them as oil slicks or look-alikes. In the past, neural network architectures typically had only three layers (input, hidden and output) as in [21], where a Multilayer Perceptron (MLP) and the Radial Basis Function (RBF) networks were used. The classification can be performed at two levels of detail: classification of pixels representing oil slicks when the number of images in the dataset is small, but they have a high resolution [22] or scenarios where the dataset contains many images [11].

More recently, many approaches based on deep learning techniques have been proposed to increase the success rate in image classification tasks. The main motivation for using deep convolutional neural networks (CNN) is their ability to extract suitable features for the task at hand, as it is very difficult to properly select the features that can allow us to discriminate between oil spills and other natural phenomena due to the similarity of their representations as dark areas in the image. In this line, Chen et al. [23] selected and optimized the PolSAR features reducing the feature dimensions used as input of the classifier to distinguish oil spill and biogenic look-alikes through layer-wise unsupervised pre-training. For this task, they use Stacked AutoEncoders (SAE, autoencoders with multiple layers) and Deep Belief Networks (DBN). In addition, Guo et al. [24] proposed a CNN to identify dark areas in SAR images as crude oil (oil slick), plant oil and oil emulsion (both look-alikes), reaching average success rates of 91% vs. the 80% of a traditional neural network. In all these works, authors used SAR imagery.

There are many oil slick detection methods that use SAR imagery as input. However, it is uncommon to find detection methods using SLAR imagery. Two recent works in this line were presented in [6,25]. The first one is based on traditional image segmentation techniques, whereas the second one uses Recurrent Neural Networks (RNN) to perform the detection. Two decades ago, Ziemke [26] already proposed a RNN using SAR images for oil spill detection, showing robustness to variations in both weather conditions and illumination changes.

Unlike the previous works using SLAR, we propose an approach that is able to detect oil slicks even in the presence of look-alikes. Our method, which is an extension of a previous study presented in [27], is focused on solving the oil-slick region segmentation problem using deep learning techniques, particularly denoising autoencoders using Convolutional Neural Networks as encoder and decoder functions.

The rest of the paper is structured as follows: Section 2 introduces background on autoencoders, Section 3 presents the proposed method, followed by the dataset description in Section 4, the evaluation in Section 5, and finally the conclusions and future work in Section 6.

## 2. Autoencoder Architecture

*Autoencoders* were proposed decades ago by Hinton and Zemel [28], and since then they have been an active research field [29]. Autoencoders consist of feed-forward neural networks trained to reconstruct their input, that is, the input and the output must be exactly the same. This problem may seem trivial as their goal is to learn the identity function f(x)=x, but, in practice, we impose some restrictions in order to force it to generate a compressed intermediate representation. This is achieved by using intermediate layers with a size smaller than the input layer. This bottleneck forces the network to extract the most representative characteristics of the sample that allow its subsequent reconstruction, thus generating a meaningful intermediate representation.

**Figure 1:**
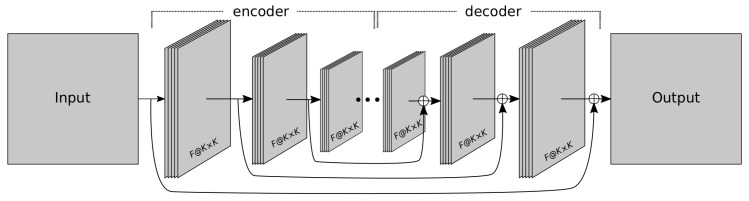
Example of a RED-Net topology. The number of layers can change according to the chosen topology. The symbol ⊕ denotes element-wise sum of feature maps. *F* represents the number of selected filters and (K×K) the size of the kernel.

Figure 1 shows a graphical scheme of an autoencoder. This type of network is divided into two parts, the first part (called the *encoder*) receives the input and creates a latent (or encoded) representation of it, and the second part (the *decoder*) takes this intermediate representation and tries to reconstruct the input. Formally speaking, given an input *x*, the network must minimize the divergence L(x,g(f(x))), where *f* and *g* represent the encoder and decoder functions, respectively. The encoder function provides a meaningful compact representation, which might be of great interest as regards feature learning or dimensionality reduction [30].

Some variations of autoencoders have been proposed in the literature to solve other kinds of problems. For example, *denoising autoencoders* [31] are an extension trained to reconstruct the input *x* from a corrupted version (usually generated using Gaussian noise) of it, denoted as $\hat{x}$. Thus, these networks are trained to minimize the divergence $L(x,g\left(f\left(\hat{x}\right)\right))$, therefore they not only focus on copying the input but also on removing the noise [31,32,33].

Autoencoders, and particularly denoising autoencoders, have been successfully used in many fields such as music, character recognition or medical image segmentation, but, in addition, they are currently being used in remote sensing to perform recognition and scene classification. For example, Zhao et al. [34] combined Stacked Autoencoder (SAE) and Extreme Machine Learning (ELM) techniques for target recognition from raw data of High-Resolution Range Profile (HRRP) acquired from three different aircraft, achieving a faster time response than other deep learning models. Other authors such as Kang et al. [35] used 23 baseline features and three-patch Local Binary Pattern (LBP) features that were cascaded and fed into an SAE for recognition of 10-class SAR targets. In addition, Liang et al. [36] presented a classification method based on Stacked Denoising Autoencoders (SDAE) in order to classify pixels of scenes (acquired from a GF-1 high resolution satellite) into forest, grass, water, crop, mountains, etc.

In this paper, we propose using autoencoders that receive as input the signal of SLAR sensors and return as output the areas detected as oil spills.

## 3. Proposed Method

Based on the idea of *denoising autoencoders*, we use a type of segmentation autoencoder as proposed in [37] but specifically designed for oil spill detection. In this case, we do not aim to learn the identity function as autoencoders do, nor an underlying error as in denoising autoencoders, but rather a codification that maintains only those input pixels that we select as relevant. This is achieved by modifying the training function so that the input is not mapped identically at the output. Instead, we train it with a ground truth of the input image pixels that we want to select. From here on, we will refer to this model as *Selectional AutoEncoder* (SelAE). The SelAE is trained to perform a function such that $s:R^{\left(w\times h\right)}\to {\left[0,1\right]}^{\left(w\times h\right)}$, or, in other words, a binary map over a w×h image that preserves the input shape and outputs the decision in the range of [0,1].

Following the autoencoder scheme, the network is divided into encoding and decoding stages, where the encoder and decoder functions can be seen as a translator between the input, the intermediate representation, and the desired segmentation. The topology of a SelAE can be quite varied. However, we have considered only convolutional models because they have been applied with great success to many kinds of problems with structured data, such as images, video, or audio, demonstrating a performance that is close (or even superior in some cases) to the human level [38].

The topology of the network consists of a series of convolutional plus *Max Pooling* layers until reaching an intermediate layer in which the encoded representation of the input is attained. It then follows a series of convolutional plus upsampling layers that generates the output image with the same input size. All layers have *Batch Normalization* [39] and *Dropout* [40], and use *ReLU* as activation function [41].

The last layer consists of a set of neurons with sigmoid activations that predict a value in the range of [0,1]. Those pixels whose selection value exceeds the *selectional* level δ—which can be seen as a threshold—are considered to belong to an oil spill, whereas the others are discarded.

In addition, in this work, we incorporate into this architecture a series of residual connections as proposed in [42]. This type of topology, called RED-Net (Very deep Residual Encoder-Decoder Network), includes residual connections from each encoding layer to its analogous decoding layer (see Figure 1), which facilitates convergence and leads to better results. Moreover, down-sampling is performed by convolutions using stride, instead of resorting to pooling layers. Up-sampling is achieved through transposed convolution layers, which perform the inverse operation to a convolution, to increase rather than decrease the resolution of the output.

We applied a *grid-search* technique [43] in order to find the network architecture with the best configuration of layers and hyperparameters (filters of each convolution, the size of the kernel, and the dropout value). The results of this experimentation are included in Section 5.4, although we anticipate the best topologies for each network in Table 1.

### 3.1. Training Stage

As autoencoders are feed-forward networks, they can be trained by using conventional optimization algorithms such as gradient descent. In this case, the tuning of the network parameters is performed by means of stochastic gradient descent [44] considering the adaptive learning rate proposed by Zeiler [45]. The loss function (usually called *reconstruction loss* in autoencoders) can be defined as the squared error between the ground truth and the generated output. In this case, we use the cross-entropy loss function to perform the optimization of the network weights during a maximum of 100 epochs, with a mini-batch size of eight samples. The training process is stopped if the loss does not decrease during 10 epochs.

**Figure 2:**
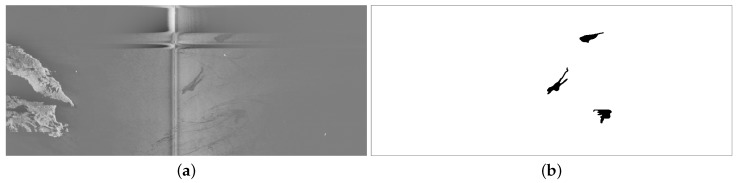
Example of a SLAR sequence from our dataset (**a**) and its corresponding ground truth (**b**) with the oil spills labeled at the pixel level. The SLAR image shows an island on the left side, a vertical zone of noise caused by junction of the signal from the two antennas of TERMA radar, and two horizontal bands of noise at the top produced by aircraft maneuvers.

In order to train the network, we generated a ground truth marking those pixels of the SLAR input images that correspond to oil spills. Figure 2 shows an example of a SLAR sequence (**a**) and its corresponding ground truth (**b**) with the oil spills labeled in black.

In this work, the network is fed with the raw data and the ground truth segmentations, so it must learn to discriminate the areas with oil spills from the rest of the data. That is, no preprocessing is performed on these images to eliminate the noise, as happens in other approaches such as in [46], nor is any post-processing done to refine the detection.

The next section details all the information about the dataset and the SLAR images used.

## 4. Dataset

In order to validate the effectiveness of the proposed method, we used a dataset containing 38 flight sequences supplied by the Spanish Maritime Safety and Rescue Agency (SASEMAR). SASEMAR is the public authority responsible for monitoring the Exclusive Economic Zones (EEZ) in Spain and its procedures are based on reports from the European Maritime Safety Agency (EMSA). The data provided by the SLAR sensor of each of these sequences was digitized in images with a resolution of 1,150×481 pixels.

**Figure 3:**
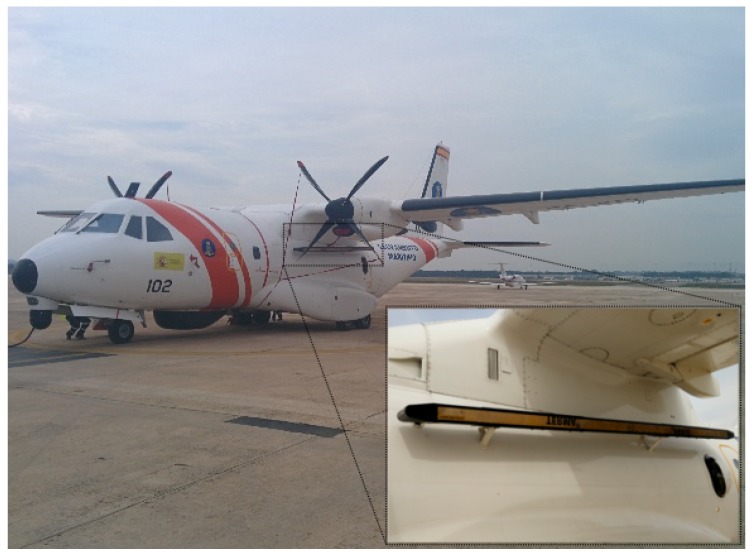
SASEMAR 102 (Variant of CN-235-300 aircraft model for search-and-rescue missions) used to obtain the SLAR sequences, manufactured by EADS-CASA.

The SLAR samples were acquired by a TERMA SLAR-9000 mounted on a variant of the EADS-CASA CN-235-300 aircraft for search-and rescue missions (see Figure 3). This aircraft model reaches a maximum cruise speed of 236 kn, a flight range of 1565 nmi and around 2700 nmi with and without payload, respectively. Its flight endurance is close to 9 h. The SLAR samples are digitalized as 8-bit integers due to the constraints of the monitoring equipment installed on the aircraft. Our autoencoder architecture uses as input these SLAR images in the same format in which they were generated by the TERMA software.

The dataset was captured by the aircraft on Spanish coasts at an approximate average altitude of 3271 feet (although the most common altitude for our missions was around 4550 feet) and with a wind speed ranging between 0 and 32 kn, the most usual being 14 kn.

As stated before, for the ground truth, we used a binary mask for each SLAR image, delimiting the pixels corresponding to oil spills. It is important to note that this labeling is performed at a pixel level since the goal is to evaluate both the detection and the precise location of the spills. This way, we can provide relevant information such as the position, the size and the shape of oil slicks in order to track them.

**Figure 4:**
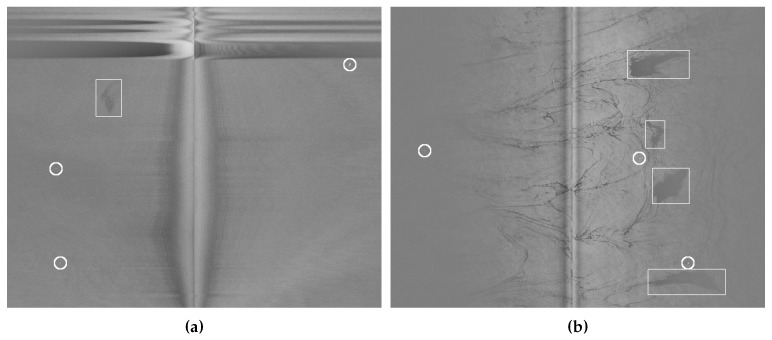

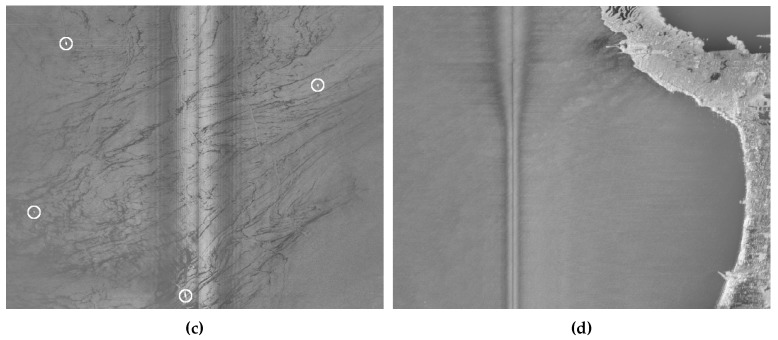
Examples of SLAR images from our dataset showing oil spills (marked with a bounding box), ships (small bright points marked with circles), look-alikes (elongated shapes in figures (**b**,**c**), the noise produced by the sensor (the central vertical band that appears in all the images) and the aircraft maneuvers (the horizontal bands that appear in the upper part of figure (**a**)), and an example of coast (on the right side of figure (**d**)). Figures (**c**,**d**) do not contain any example of oil spills; however, they have other artifacts that can lead to confusion.

Figure 4 shows four examples of SLAR images from our dataset. They contain several oil spills (marked with a bounding box in Figure 4a,b, along with other types of artifacts such as boats (small bright points), coast (Figure 4d), look-alikes, and noise. Figure 4b,c contain many examples of look-alikes, with elongated shapes that are very similar to those of actual spills. All figures show a central band of noise, which is produced by the union of the information from the two SLAR sensors. In addition, the upper part of Figure 4a,d shows the noise generated by turning maneuvers of the airplane and the effect produced when the aircraft changes its altitude, respectively.

From the 38 flight sequences, 22 contain examples of oil spills and 4 of look-alikes. Within these examples, the spots only represent 0.32% of the pixels in the image, which creates a very unbalanced dataset. To evaluate the method properly in the presence of unbalanced data, we use the $F_{1}$ and in addition other metrics described in Section 5.1.

In all the experiments, we used an *n*-fold cross validation (with n=5), which yields a better Monte Carlo estimate than when solely performing the tests with a single random partition [47]. Our dataset was consequently divided into *n* mutually exclusive sub-sets, using the data of each flight sequence only in one partition and maintaining the percentage of samples for each class. For each fold, we used one of the partitions for test (20% of the samples) and the rest for training (80%).

For tuning the hyperparameters (see Section 5.4), the training partitions were divided into two, assigning 10% of these samples for validation and the rest (70%) for training. The classifier was trained and evaluated *n* times using these sets, after which the average results plus the standard deviation σ were reported.

## 5. Evaluation

This section shows the experiments performed. First, we describe the metrics used for the evaluation, followed by the augmentation methodology and the type of normalization applied. Then we present the best hyperparameters found by the grid-search process and, finally, the results obtained by the proposed method.

The following experiments were made on an SGI ICE XA system (Cirrus UK National Tier-2 High Performance Computing Service at EPCC) with two 2.1 GHz, 18-core Intel(R) Xeon E5-2695 (Broadwell) and 256 GB RAM. The computational resources from this machine were mainly exploited to parallelize the grid-search process in order to explore several network configurations.

### 5.1. Evaluation Metrics

Three evaluation metrics widely used for this kind of tasks were chosen to evaluate the performance of the proposed method: Precision, Recall, and $F_{1}$, which can be defined as:

(1) $$\begin{array}{cc} \mathrm{Precision} & =\frac{\mathrm{TP}}{\mathrm{TP}+\mathrm{FP}}, \end{array}$$

(2) $$\begin{array}{cc} \mathrm{Recall} & =\frac{\mathrm{TP}}{\mathrm{TP}+\mathrm{FN}}, \end{array}$$

(3) $$\begin{array}{cc} F_{1} & =\frac{2\cdot \mathrm{TP}}{2\cdot \mathrm{TP}+\mathrm{FN}+\mathrm{FP}}, \end{array}$$

where TP (True Positives) denotes the number of correctly detected targets (pixels), FN (False Negatives) the number of non-detected or missed targets, and FP (False Positives or false alarms) the number of incorrectly detected targets.

It should be noted that the $F_{1}$ metric is suitable for unbalanced datasets, but it is not the most adequate for this task since it measures the precision of the results at the pixel level but not whether the algorithm has detected the spill or not. For this reason, we also use the Intersection over Union (IoU) for evaluation, measuring whether the algorithm correctly detects all the spills present in the image and also how well it detects their size and location.

In order to calculate the IoU, we map each object proposal (*p*) to the ground-truth (*g*) bounding box with which it has a maximum IoU overlap. Bounding boxes are calculated to include the groups of pixels (or *blobs*) marked as 1 in the network output after applying the selectional threshold or in the ground-truth images. A detection is considered as TP if the area of overlap ($a_{o}$) ratio between the predicted bounding box ($B_{p}$) and the ground-truth bounding box ($B_{g}$) exceeds a certain threshold (λ) according to the following equation:

(4) $$\begin{array}{cc} a_{o} & =\frac{area(B_{p}\cap B_{g})}{area(B_{p}\cup B_{g})},\\ TP & =a_{o}>\lambda , \end{array}$$

where $area(B_{p}\cap B_{g})$ depicts the intersection between the object proposal and the ground truth bounding box, and $area(B_{p}\cup B_{g})$ depicts its union. By convention, we use a threshold of λ=0.5 to select a TP candidate.

### 5.2. Normalization

Initially, we conducted an experiment to determine the best type of normalization for the task at hand. The literature cites different ways to normalize the data to feed a network [48,49], but the most appropriate technique depends on the particular problem. The most common normalization methods are:

(5) $$\begin{array}{cc} Z_{standard} & =\frac{M-mean(M)}{std(M)}, \end{array}$$

(6) $$\begin{array}{cc} Z_{min-max} & =\frac{M-min(M)}{max(M)-min(M)}, \end{array}$$

(7) $$\begin{array}{cc} Z_{mean} & =M-mean(M), \end{array}$$

(8) $$\begin{array}{cc} Z_{norm} & =M/255, \end{array}$$

where *M* is the input matrix containing the raw image pixels from the training set. For the normalization of the test set, we used the same mean, deviation, max, and min values calculated for the training set. It is also important to note that the range of values obtained depends on the equation used; however, this is not an issue since the configuration of the network allows it, and, as stated before, this can lead to a better result.

We evaluated these types of normalization on the two networks, including the option of not normalizing the data. For this, we considered a base configuration (with 32 filters per layer, a kernel size of 3×3, a dropout of 0.25, and a *selectional* threshold δ of 0.5), and then we varied the input size (subsampling the input images to 128 × 128 px and 256 × 256 px) and the number of hidden layers of each network (from four to eight), in order to obtain a statistically significant average result. The networks were trained using a data augmentation of 20 (see Section 5.3) on the training set, and, for the evaluation, we used the validation set.

The results of this experiment (in terms of $F_{1}$, see Equation (3)) are shown in Table 2, where each cell shows the average of 30 experiments (six network configurations per five folds). As can be seen, the best $F_{1}$ for the two types of networks are obtained using the standard normalization, followed by the mean norm. The type of data normalization considerably affects the result obtained, since the differences in some cases reach up to 25%. For this reason, in the following experiments, we use the standard normalization.

### 5.3. Data Augmentation

Data augmentation is applied in order to artificially increase the size of the training set [49,50]. As the experimental results show, augmentation systematically improves the accuracy.

To this end, we randomly applied different types of transformations on the original images, including horizontal and vertical flips, zoom (in the range [0.5, 1.5] times the size of the image), rotations (in the range [−10${}^{\circ }$, 10${}^{\circ }$]), and shear (between [−0.2${}^{\circ }$, 0.2${}^{\circ }$]).

In order to evaluate the improvement obtained with this augmentation process, we carried out an experiment in which we gradually increased the number of random transformations applied to each image from our training set, and evaluated it using the validation set. As before, we performed this experiment for both architectures fixing the configuration to 32 filters per layer, a kernel size of 3×3, a dropout of 0.25, and a *selectional* threshold δ of 0.5, and only varying the input size (subsampling the input images to 128 × 128 px and 256 × 256 px) and the number of hidden layers of each network (from four to eight). The input data was normalized using standard normalization.

**Figure 5:**
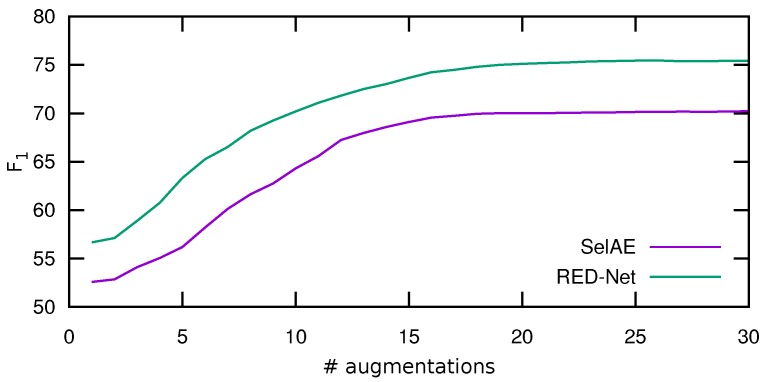
Average results of the data augmentation process. The horizontal axis represents the number of augmentations and the vertical axis the average $F_{1}$ (in percentage) obtained for each of the networks.

Figure 5 shows the average results of such experiment, where the horizontal axis indicates the augmentation size and the vertical axis the $F_{1}$ obtained. As can be seen, for the two models evaluated, the highest improvement is obtained at the beginning, after which the results begin to stabilize and stop improving after 20 augmentations. For this reason, in the following, we set to this value the number of augmentations applied to each image.

### 5.4. Hyperparameters Evaluation

In order to select the best hyperparameters for the two types of CNN evaluated, we have performed a *grid-search* [43] using the training and validation sets. The configurations evaluated include variations in the network input size (from 32 px to 512 px per side), in the number of layers (from four to eight), the number of filters (between 16 and 128), the kernel size (between three and seven), the percentage of dropout (from 0 to 0.5), and the *selectional* threshold δ (between 0 and 1). Overall, 6480 experiments were made, using 1296 configurations × 5 folds. In all cases, we applied an augmentation of 20 and the standard normalization.

**Figure 6:**
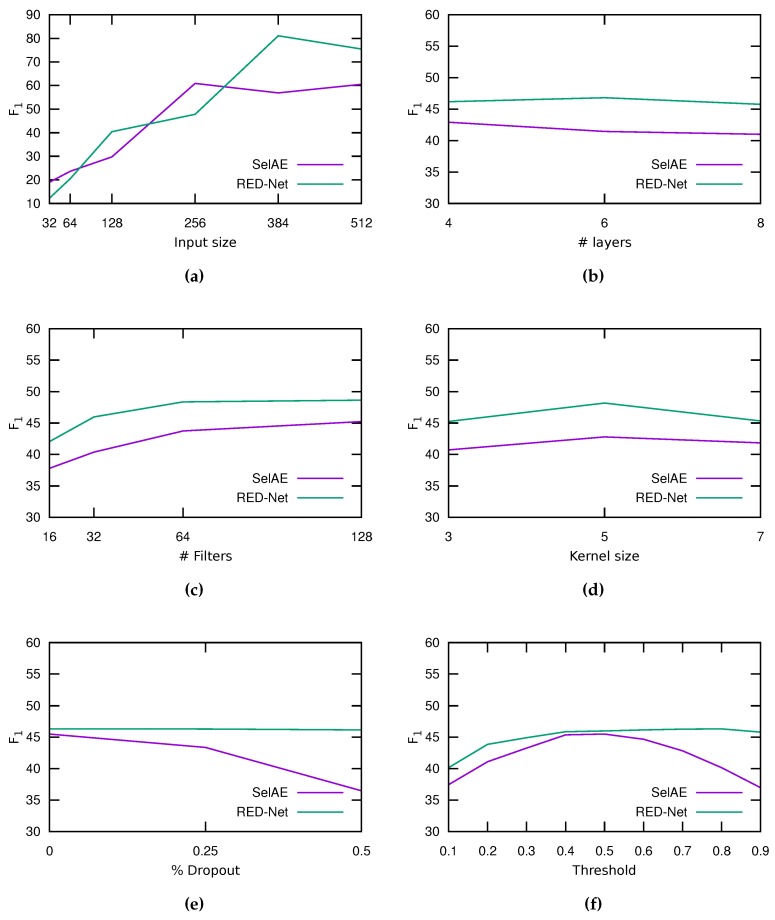
Average $F_{1}$ (%) of the grid-search process when varying (**a**) the input image size; (**b**) the number of layers; (**c**) the number of filters per layer; (**d**) the kernel size of the convolutional filters; (**e**) the percentage of dropout, and (**f**) the *selectional* threshold δ.

Figure 6 shows the results of this experiment. The average $F_{1}$ when varying the input size is shown in Figure 6a. As can be seen, larger inputs are beneficial for this task. The SelAE architecture obtains the higher $F_{1}$ with a 256 × 256 px size, whereas the most suitable size for RED-Net is 384 × 384 px. Figure 6b shows the results when varying the number of layers. The SelAE architecture obtains the best $F_{1}$ with four layers, whereas RED-Net requires six layers. This may happen because pooling layers lose information for the reconstruction, whereas RED-Net mitigates this loss through residual layers. Figure 6c shows the average $F_{1}$ obtained when varying the number of filters per layer. Using more filters increases the $F_{1}$, and this improvement is noticeable from 16 until 64 filters, only increasing marginally with 128 filters. Figure 6d shows the average $F_{1}$ for the three kernel sizes evaluated, and both architectures obtained the best results with 5 × 5 filters. Figure 6e shows the average $F_{1}$ obtained by varying the dropout percentage applied to each layer. The best result for both architectures in this experiment was obtained without using dropout. The RED-Net results remain stable, but they slightly worsen when increasing the dropout, whereas, with SelAE, the $F_{1}$ is significantly lower when dropout grows. Finally, Figure 6f shows the result by varying the *selectional* threshold δ. RED-Net remains fairly stable to changes in this value, obtaining its maximum for a threshold of 0.8. SelAE seems to be more affected by changes, obtaining better results with an intermediate threshold of 0.5.

In conclusion, the final architecture chosen for each network is with 128 filters with 5 × 5 size and without dropout. The SelAE uses an input size of 256 × 256 px, 4 layers, and a threshold of 0.5, whereas RED-Net uses 384 × 384 px with six layers and a threshold of 0.8. Table 1 shows a summary of the topologies that were eventually chosen.

### 5.5. Results

Once the best configuration and parameter settings for each network were selected, we evaluated the results using the different metrics. Moreover, we compared these results with three state-of-the-art methods for oil slick segmentation in SLAR images:

- “*Graph-based method*” [51]: It is an adaptation for SLAR images of the method proposed in [52]. It uses progressive intensity gradients for extracting regions with variable intensity distribution.
- “*JSEG method*” [51]: It is also an extension to SLAR images of a previous work [53], where the input image is quantized according to the number of regions to be segmented. Pixel intensities are replaced by the quantized label building a class-map called J-image. Later, a region-growing technique is used to segment the J-image.
- “*Segmentation guided by saliency maps (SegSM)*” [6]: It first applies a pre-process of the noise caused by aircraft movements using Gabor filters and Hough Transform. Then, the saliency map is computed and used as seeds of a region-growing process that segments the regions that represent oil slicks.

Details regarding the implementation and the parameters used in these methods can be found in the corresponding references.

Table 3 shows the final result obtained with the proposed approach as well as the comparison with the state-of-the-art methods using the test set for the evaluation. It should be noted that the test set had not been used in previous experiments to avoid adjusting the network architecture for this set.

The best results were obtained in all cases using the RED-Net architecture, which shows a higher $F_{1}$ (see Equation (3)) for all the tested images. On the one hand, the best RED-Net configuration improves by up to 3.7% the $F_{1}$ of the SelAE autoencoder, and, between 37% and 64%, the other methods of the state of the art. The SegSM method has a high precision and a low recall, which indicates that it accurately detects some parts of the spills but producing many FN. On the other hand, both JSEG and Graph-based methods have a high recall and a low precision, since, in this case, they are producing many false positives. The proposed method obtains a more balanced result in the detection of oil-spill pixels. This fact can be confirmed by looking at the IoU metric (see Equation (4)), where RED-Net also improved significantly the results with respect to the other methods, which indicates a better precision in the detection of the shape and the position of the oil slicks.

**Figure 7:**
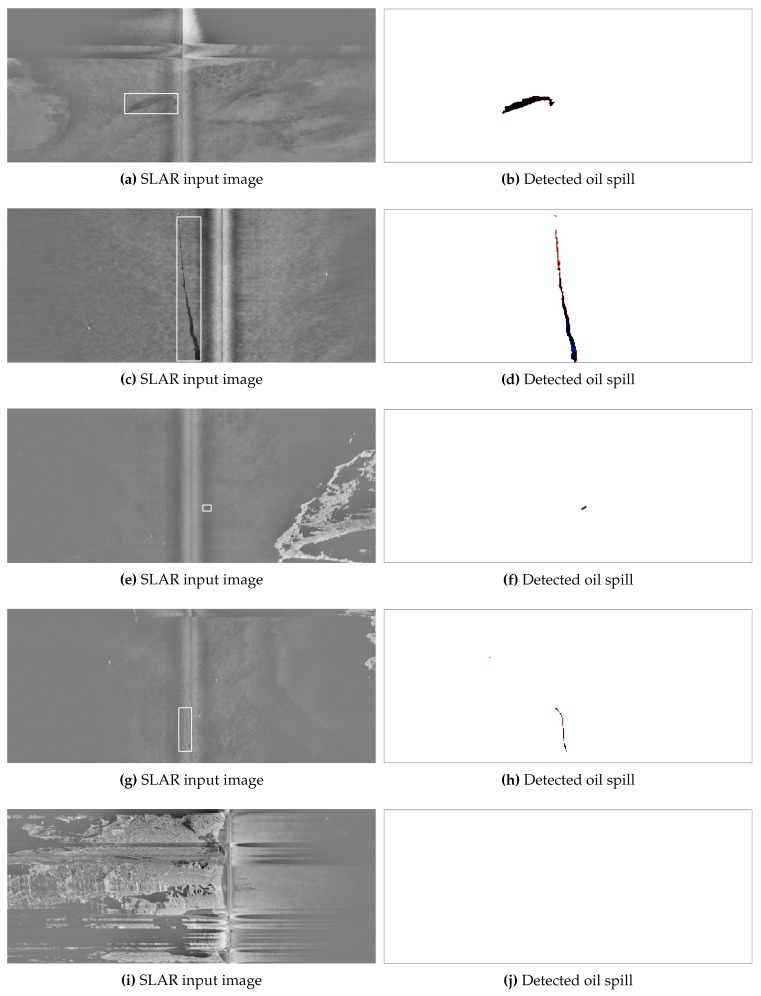
Results of processing five SLAR input images. The first column (**a**,**c**,**e**,**g**,**i**) shows the original SLAR images, where oil spills are marked with a bounding box; the second column (**b**,**d**,**f**,**h**,**j**) shows the detection results. White and black areas depict correct detections of sea and oil spills, respectively, and red and blue pixels (hard to see because they are few) depict FP and FN of oil spills, respectively.

Figure 7 shows a graphic representation of the results obtained with the best approach, i.e., the RED-Net model. The first column of images shows the original input SLAR images (oil spills are marked with a bounding box), and the second column shows the prediction of the network. In the images of the second column, white and black areas depict correct detections of sea and oil spills, respectively, and red and blue pixels depict FP and FN of oil spills, respectively.

These figures help to visualize the accuracy of the proposed model and to understand where the errors of each target class occur. As can be seen, wrong detections are typically made only at the contours of the oil spills.

Figure 7a shows that the proposed method correctly detects the spill even in the presence of noise due to look-alikes (biological origin and weather conditions). In Figure 7c, we can see a larger spill produced by a ship emptying its bilge tanks. This spill is correctly detected and there are only few mistakes at the edges. Figure 7e contains coast, but the method does not create false positives and it correctly detects just one small spill at the center. Figure 7g also shows a coast section in the upper-right part, and, in this case, the image contains an airplane turn. In this example, even when the spill is located at the center of the noise, the method is able to correctly perform the detection. Finally, the last example in Figure 7i shows an image with high noise (caused by aircraft movements), including coast at the left side and without any spill. As can be seen, the method correctly concludes that the image does not contain any spill.

## 6. Conclusions

In this work, we present a method that uses deep convolutional autoencoders for the detection of oil spills from SLAR imagery. Two different network topologies have been analyzed, conducting extensive experiments to get the best type of data normalization, to know the impact of data augmentation on the results, and to obtain the most suitable hyperparameters for both networks.

A dataset with a total of 28 flight sequences was gathered on Spanish coasts using TERMA SLAR radar, with labeled the ground-truth in order to train both selectional autoencoders and RED-Nets. It is composed of oil spills acquired in a wide variety of sea conditions dependent on weather (i.e., wind speed) and geographic location as well as of flight conditions such as altitude and type of motion.

The proposed approach is able to segment accurately oil slicks despite the presence of other dark spots such as biogenic look-alikes, low wind, which also introduces a lot of look-alikes, and noise due to bad radar measurements caused by the aircraft maneuvers. Results show that the RED-Net achieves an excellent $F_{1}$ of 93.01% when evaluating the obtained segmentation at the pixel level. In addition, by analyzing the precision of the regions found using the Intersection over Union (IoU) metric, the proposed method correctly detects 100% of the oil spills, even in the presence of artifacts and noise caused by the aircraft maneuvers, in different weather conditions and with the presence of look-alikes.

Future work includes increasing the dataset size by adding more labeled samples from additional missions. In addition, Generative Adversarial Networks (GAN) such as Pix2Pix [54] could be used to deal with a reduced dataset by generating synthetic samples. In addition, the detected oil slick locations could be used to initialize oil spill models for better oil spill prediction and response [55]. A study correlating the $F_{1}$ score with the wind speed or weather conditions could also be useful to understand to what extent the effectiveness of the proposed method depends on these factors.

References

## Figures and Tables

**Table 1:** Best architectures found after the grid-search process.

Autoencoder Type:	SelAE	RED-Net
**Input image size:**	256 × 256 px	384 × 384 px
**Number of layers:**	4	6
**Residual connections:**	No	Yes
**Filters per layer:**	128	128
**Kernel size:**	5 × 5	5 × 5
**Down-sampling:**	MaxPool (2 × 2)	Stride (2 × 2)
**Dropout (%):**	0	0
***Selectional*** **threshold** δ**:**	0.5	0.8

**Table 2:** Average $F_{1}$ (%) plus σ when applying different types of normalization on the input data, and without normalization.

	None	$Z_{Standard}$	$Z_{min-max}$	$Z_{mean}$	$Z_{norm}$
**SelAE**	54.33 ± 2.23	**70.02** ± 1.26	44.65 ± 3.14	69.84 ± 1.67	44.10 ± 3.57
**RED-Net**	65.25 ± 1.97	**75.12** ± 1.07	53.66 ± 2.75	74.91 ± 1.35	59.67 ± 2.91

**Table 3:** Evaluation results including the standard deviation for the two architectures using the chosen parameters after grid-search.

Model	Precision	Recall	$F_{1}$	IoU
**Graph-based**	32.99 ± 1.62	97.25 ± 0.33	48.28 ± 1.87	32.55 ± 0.16
**JSEG**	17.04 ± 0.32	92.58 ± 0.25	28.73 ± 0.46	16.50 ± 0.35
**SegSM**	98.54 ± 0.27	39.55 ± 1.21	55.78 ± 1.18	87.33 ± 0.51
**SelAE**	89.64 ± 0.95	88.99 ± 0.91	89.31 ± 0.93	92.14 ± 7.21
**RED-Net**	93.12 ± 0.86	92.92 ± 0.84	**93.01**± 0.85	100.00 ± 0.00
